# Perioperative Antimicrobial Prophylaxis in Elective and High-Risk Laparoscopic Cholecystectomy: A Narrative Review

**DOI:** 10.7759/cureus.101861

**Published:** 2026-01-19

**Authors:** Narayan Khanal, Kyle Green

**Affiliations:** 1 General Surgery, Albury Wodonga Health, Albury, AUS; 2 General Surgery, Wagga Wagga Base Hospital, Wagga Wagga, AUS

**Keywords:** antimicrobial stewardship program, high-risk laparoscopic cholecystectomy, laparoscopic cholecystectomy, perioperative antibiotic prophylaxis, prophylactic antibiotic use, surgical site infections (ssis)

## Abstract

Laparoscopic cholecystectomy (LC) is one of the most frequently performed general surgical procedures. Surgical site infection (SSI) rates are low in low-risk elective cases, yet practice variation in perioperative antimicrobial prophylaxis remains considerable. Questions persist regarding the necessity of prophylaxis. Optimising antimicrobial use in LC may represent an area of interest for antimicrobial stewardship within general surgery. A narrative review of literature published between 2010 and 2025 was conducted using MEDLINE, Embase and Cochrane databases. Randomised controlled trials, observational studies, meta-analyses and international guidelines evaluating antimicrobial use for LC were included. This review synthesised current evidence regarding SSI risk in elective and high-risk cases, compared major guidelines and explored stewardship implications. Across multiple randomised trials and meta-analyses, routine antimicrobial prophylaxis has not consistently been shown to confer a clinically meaningful reduction in SSI rates for low-risk elective LC, with reported benefits limited by heterogeneity, small absolute risk reductions and variable study quality. In high-risk LC, particularly acute cholecystitis and cases involving bile contamination or severe inflammation, baseline SSI risk is higher; however, evidence does not consistently demonstrate benefit from perioperative or postoperative antibiotics. Data for other high-risk subgroups, including patients with diabetes, obesity, immunosuppression or prolonged operative time, are limited and largely derived from observational studies. In high-risk cases, evidence remains insufficient to draw definitive conclusions, contributing to ongoing practice variation. Given the implications for antimicrobial resistance, healthcare costs and patient safety, further high-quality prospective studies focusing on clearly defined high-risk subgroups are required to inform guideline development and optimise antimicrobial stewardship. As a narrative review, this paper aims to summarise and contextualise existing evidence rather than provide definitive practice recommendations.

## Introduction and background

Laparoscopic cholecystectomy (LC) is one of the most commonly performed general surgical procedures for symptomatic gallbladder disease [1,2]. Since its introduction in the late 1980s, the laparoscopic approach has largely replaced open surgery due to the advantages of reduced postoperative pain, shorter hospital stays and lower overall complication rates [2,3]. The procedure represents a substantial proportion of the operative workload for general surgeons and is a key area for optimising antimicrobial stewardship. Surgical site infections (SSIs) account for a large proportion of healthcare-associated infections and contribute to increased morbidity, readmission rates, healthcare costs and overall antimicrobial use [4]. Although the absolute risk of SSIs after elective LC is low, the high volume of procedures performed means that even modest improvements in perioperative antimicrobial practice may yield substantial benefits at a population level [5]. A single dose of preoperative antibiotic prophylaxis has traditionally been used to minimise infection risks; however, international practice remains inconsistent [5-8].

The laparoscopic technique for cholecystectomy has dramatically reduced infection risk [9]. This shift has sparked an ongoing debate regarding the necessity of prophylaxis in low-risk elective cases, with some studies suggesting that antibiotics may be omitted entirely without increasing SSI rates [5,10,11]. Practice variation remains substantial; survey data suggest that approximately 30% of surgeons routinely administer prophylaxis for elective LC, and a further 60% use it selectively [12]. In contrast, the term “high-risk” LC encompasses a heterogeneous group of clinical scenarios that differ fundamentally in baseline risk and in the intended role of antibiotics. These include emergency surgery for acute cholecystitis (AC) and gallbladder empyema, where antibiotics may be administered as treatment rather than prophylaxis, and where infectious complications extend beyond SSIs; elective procedures in patients with recognised risk factors such as advanced age, obesity, diabetes, immunosuppression and prolonged operative time, which may prompt escalation beyond standard prophylaxis [13]. Conflation of these distinct contexts complicates the interpretation of the literature and contributes to wide variation in clinical practice. Inappropriate antimicrobial use in surgery is a significant driver of antimicrobial resistance (AMR) [14]. Excessive or prolonged prophylaxis contributes to selection pressure for resistant organisms, increases Clostridioides difficile risk, and exposes patients to unnecessary adverse drug effects [15]. In the context of global AMR concerns, perioperative antibiotic prescribing in general surgery is an important target for stewardship interventions.

This narrative review synthesises evidence regarding perioperative antimicrobial prophylaxis in LC across elective and higher risk clinical contexts. It examines SSI risk and evaluates evidence for prophylaxis effectiveness in low-risk and heterogeneous higher-risk settings. It also compares major international guidelines to identify areas of alignment and divergence. Finally, it discusses antimicrobial stewardship implications and highlights areas where further research is needed to optimise practice.

## Review

Methods

Study Design

This study was conducted as a narrative review of the existing literature evaluating perioperative antimicrobial use in LC. A narrative approach was deliberately chosen to enable broad synthesis and contextual interpretation of heterogeneous evidence, including systematic reviews, meta-analyses, randomised trials, observational studies and international guideline documents. Given the diversity of study designs, patient populations, clinical contexts and outcome definitions across the literature, the intent of this review was interpretive rather than quantitative, and no formal meta-analysis, risk-of-bias stratification or certainty grading was undertaken. This review was designed to explore and contextualise three overarching clinical themes, namely the existing evidence for antimicrobial prophylaxis in elective and higher-risk LC, variation in SSI risk across clinical contexts and areas of alignment and divergence among international guideline recommendations.

Literature Search

A literature search was performed across the electronic databases of MEDLINE (via PubMed), Embase and Cochrane Library to identify representative and influential publications relevant to perioperative antimicrobial prophylaxis in LC. The search included studies published between January 2010 and December 2025, reflecting contemporary laparoscopic practice, with selective inclusion of earlier landmark studies where findings remain influential or relevant. Search terms included combinations of keywords related to LC, antimicrobial prophylaxis, SSI, AC, bile spillage and antimicrobial stewardship. Reference lists of relevant systematic reviews, meta-analyses and international guidelines documents were manually screened to identify additional pertinent studies.

Consistent with the narrative design, the literature search was conducted to capture key studies and guideline statements relevant to clinical practice rather than to achieve exhaustive or reproducible retrieval. Formal record counts, screening workflows, study flow diagrams and structured risk-of-bias or certainty grading frameworks (e.g. ROB2, ROBINS-I, GRADE) were therefore not applied. The findings should be interpreted as a contextual overview of the existing evidence base rather than as a comprehensive or graded evidence synthesis.

Inclusion and Exclusion Criteria

Studies were included if they met one or more of the following criteria: investigated perioperative antibiotic prophylaxis in LC; reported SSI rates or comparative outcomes; published RCTs, cohort studies, case-control studies, systematic reviews and meta-analyses; relevant international clinical guidelines. The exclusion criteria were non-English publications; case reports or case series with fewer than 20 patients; studies focusing exclusively on open cholecystectomy; studies limited to paediatric populations; animal studies. For the purposes of this review, “high-risk” was structured into three different groups. These three groups are as follows: AC, elective with patient-level risk factors: diabetes mellitus; obesity; older age (>70 years); immunosuppression and prolonged operative time (>90 minutes) and intraoperative contamination events.

Ethics approval was not required as this review utilised publicly available data and did not involve patient-identifiable information, or institutional data collection.

Evidence

The reported incidence of SSIs following LC is low (0.3% - 4.0%) compared to open surgery (1.1% - 8.4%), demonstrating improvements in minimally invasive techniques, perioperative care and infection prevention strategies [16]. The risk of deep or organ-space infection is particularly rare (approx. 0.20%) in elective LC, with the majority of SSIs manifesting as superficial incisional infections [17]. The reduced SSI rate in laparoscopic surgery is attributed to smaller incision size, limited tissue trauma, reduced operative exposure and shorter operative times [18]. Risk factors for SSIs in LC include older age, obesity, diabetes, prolonged operative duration, emergency surgery, AC, bile spillage and conversion to open surgery [19]. Other infectious complications are also possible, particularly in patients with AC, and include infectious collections or abscess formation and sepsis. Although infectious complications remain relatively uncommon, even low complication rates translate to significant absolute numbers given the volume of procedures performed. Infectious complications are associated with longer hospital stays, increased antibiotic exposure, higher readmission rates and increased healthcare costs.

Prophylactic Antibiotics in Low-Risk Laparoscopic Cholecystectomy

Multiple randomised controlled trials (RCTs) and meta-analyses have evaluated the role of prophylactic antibiotics in elective low-risk LC. Overall, the preponderance of contemporary evidence shows no statistically significant reduction in SSI rates with routine prophylaxis in low-risk LC [11,18,20,21]. A small number of studies have demonstrated the benefit of using prophylactic antibiotics and their effectiveness in reducing SSIs even in low-risk LC [22,23], however, with a very modest absolute reduction in SSIs with antibiotics use. A Cochrane systematic review reported no statistical difference in SSI rates between patients receiving antibiotics and those receiving a placebo for elective LC [24]. More recent systematic reviews and meta-analyses incorporating larger datasets and improved methodology have reinforced these findings, with several concluding that there is no clinical benefit with routine antibiotic prophylaxis in low-risk cases [25]. Despite this evidence, antibiotic use remains widespread in this group, with surveys indicating that approximately 30% of surgeons always use antibiotic prophylaxis and a further 63% selectively use it in elective cases [12]. Therefore, optimisation of perioperative antimicrobial practice remains clinically relevant. Table 1 summarises key randomised trials and meta-analyses evaluating prophylactic antibiotics in low-risk elective LC, demonstrating largely consistent findings of no clinically meaningful reduction in SSI rates.

**Table 1:** Summary of studies assessing the benefit of prophylactic antibiotic use for SSIs in elective low-risk LC CI: confidence interval; OR: odds ratio; RCT: randomised controlled trial; SSI: surgical site infection; LC: laparoscopic cholecystectomy

Author	Year	Study type	Sample	Key findings (antibiotics vs no antibiotics groups)
Pasquali et al. [11]	2016	Meta-analysis	19 RCTs N = 5,259	Antibiotics did not significantly reduce the risk of SSI (2.4% vs 3.2%; RR 0.81, 95% CI, 0.58 to 1.13; p = 0.21).
Sarkut et al. [18]	2017	Randomised controlled trial	N = 570	There was no statistical difference for SSI between antibiotics and non-antibiotics groups (1.04% vs 1.5%; p = 1.00).
Darzi et al. [19]	2016	Randomised controlled trial	N = 434	No significant differences between the two groups in the incidences of postoperative infection (1.7% vs 2.0%; p = 0.99).
Chong et al. [21]	2015	Prospective comparative clinical study	N = 471	The incidence of SSI was similar for the two groups, with no statistical difference (1.79% vs 1.56%; p = 0.973).
Liang et al. [22]	2016	Systematic review and meta-analysis	21 RCTs N = 5,207	Antibiotic prophylaxis reduced the incidence of SSI (risk ratio 0.61, 95% confidence interval 0.45 to 0.82; p = 0.001)
Sajid et al. [23]	2018	Systematic review and meta-analysis	25 RCTs N = 6,138	No statistically significant difference, odds ratio 0.75 (95% CI, 0.52–1.07); p = 0.11.
Sanabria et al. [24]	2010	Cochrane systematic review	11 RCTs N = 1,664	No statistically significant differences between antibiotic prophylaxis and no prophylaxis in the proportion of SSI, odds ratio 0.87 (95% CI 0.49 to 1.54).
Gomez-Ospina et al. [25]	2018	Systematic review and network meta-analysis	18 qualitative and quantitative studies N = 4,087	No significant differences were found when comparing antibiotics vs placebo/no intervention (risk difference -0.00 (95% CI − 0.01 to 0.01).

Antibiotics in High-Risk Laparoscopic Cholecystectomy

High-risk LC differs markedly from routine elective LC in both pathophysiology and baseline risk profile, comprising a heterogeneous patient cohort. Conditions such as AC, gallbladder empyema, gangrenous disease, choledocholithiasis with duct instrumentation, bile spillage and significant patient-level comorbidity result in higher rates of bactibilia, local contamination and systemic inflammation. As a result, the potential value of antimicrobial prophylaxis is greatest in this cohort, who also have increased infectious complications when compared to low-risk patients [16,26]. Given this heterogeneity, this review will consider AC, intraoperative contamination events, and elective LC with patient-level risk factors separately [16,19].

Perioperative Antimicrobial Use in Acute Cholecystitis

AC is one of the most common emergency general surgery presentations and the most widely studied high-risk indications for antimicrobial use. The nature of emergency surgery, potential rupture, and the effects of acute inflammation increase the likelihood of postoperative infectious complications, including SSIs [16,19]. While antibiotics are commonly used as a bridge to surgery or for surgical antimicrobial prophylaxis, they may also be employed as a therapeutic non-operative strategy to manage AC, usually in patients where operative management is thought to be unfavourable due to patient comorbidity, or even preference. Compared to elective LC, acute presentations are associated with increased SSI rates, higher conversion to open surgery and greater incidence of bactibilia [16,19]. Given that AC is primarily an inflammatory disease resulting from the obstruction of the cystic duct in 90-95% of cases, with only 20% of patients developing infectious complications [27], the utility of antimicrobials may need to be carefully considered. The presence of gangrenous disease or empyema increases the presence of bactibilia to between 50 and 70% [19], increasing the risk of superficial SSIs, deep infection and postoperative sepsis. The most common postoperative complication observed after AC is SSI [28]. However, unlike in elective LC, the potential for infectious complications beyond SSIs is more pronounced in AC, including deep infections, empyema, and sepsis.

A comparatively small number of RCTs have evaluated the impact of antimicrobial use on AC compared to elective LC. These have demonstrated conflicting results. A meta-analysis comprising 781 patients did not demonstrate a statistically significant reduction in infectious complications for low and moderate-severity AC, as defined by the Tokyo Guidelines [29], when single-dose pre-operative antimicrobial prophylaxis was used [30]. A further, more recent, meta-analysis explored perioperative antimicrobial prophylaxis (pre or postoperative) in a similar population, combining 1747 patients [31]. This also failed to show any benefit in reducing infectious complications regardless of whether antibiotics were used before or after surgery. Postoperative antimicrobial use has also been examined, with one systematic review and meta-analysis showing no added benefit in continuing antibiotics postoperatively [32]. While the most recent and largest analysis by Wang et al., which incorporated all patients undergoing LC not limited to acute cholecystectomy, did affirm benefit for use in elective low-risk LC, this was not the case for subgroup analysis in patients with AC [33]. This is an interesting finding, particularly considering the increased risk of infectious complications and bactibilia in AC compared to low-risk cases, but nevertheless confirms the need for further investigation in this group before conclusions can be drawn.

Elective Laparoscopic Cholecystectomy With Patient-Level Risk Factors

Several risk factors increase infection rates in surgical patients. Diabetes mellitus, obesity, advanced age, immunosuppression and prolonged operative duration are repeatedly identified as independent predictors of SSIs following LC in observational studies [16,26,34]. However, no RCTs have evaluated whether antimicrobial use reduces postoperative infectious complications specifically in patients with these isolated risk factors. A small number of RCTs have performed subgroup analysis on patients with these risk factors; however, this is limited to only AC, with none having investigated individual risk factors in elective LC (patients without AC). A RCT by Negi et al. investigated high-risk patients (including those with AC) but did not perform subgroup analysis on individual categories due to the low number of SSIs overall [35]. This study did not appropriately randomise subjects. Another single-surgeon study comparing single-dose antibiotic prophylaxis to no antibiotics on all patients undergoing LC regardless of patients' risk status was conducted, but there were no reported SSIs in either group, complicating subgroup analysis for high-risk patients (Table 2) [36]. Both studies are of low-quality evidence with significant design weaknesses, highlighting the necessity of further large, multicentre, robust investigations into the outcomes associated with omitting antimicrobial prophylaxis in high-risk subgroups undergoing LC.

**Table 2:** Summary of studies assessing benefits of prophylactic antibiotic use for SSIs in high-risk LC CI: confidence interval; IC: infective complication; LC: laparoscopic cholecystectomy; MD: mean difference; OR: odds ratio; RCT: randomised controlled trial; RR: relative risk; SSI: surgical site infection

Author	Year	Study type	Sample (prophylaxis vs no prophylaxis)	Key findings (antibiotics vs no antibiotics groups)
Singh et al. [30]	2023	Systematic review and meta-analysis	3 RCTs N = 781	There was no reduction in IC when single-dose preoperative antibiotics were given before LC for mild to moderate cholecystitis (RR = 0.69; 95% CI: 0.46–1.03; p = 0.07).
Elkasaby et al. [31]	2024	Systematic review and meta-analysis	7 RCTs N = 1,747	No differences were found in IC (RR = 0.84; 95% CI: 0.63-1.12; p = 0.23), SSI (RR = 0.79; 95% CI: 0.56-1.12, p = 0.19), or readmission (RR = 0.69; 95% CI: 0.43-1.11; p = 0.13).
Hajibandeh et al. [32]	2019	Systematic review and meta-analysis	4 RCTs N = 953	No difference in IC (OR = 0.94, 95% CI = 0.62 to 1.44, p = 0.79), SSI (OR = 1.13, 95% CI = 0.58 to 2.18, p = 0.72), postoperative morbidity (OR = 0.93, 95% = CI = 0.66 to 1.32, p = 0.70), length of hospital stay (MD = 0.78, 95% CI = −0.55 to 2.10, p = 0.25), or readmission (OR = 0.87, 95% CI = 0.42 to 1.81, p = 0.70) were found when extended postoperative antibiotics versus no postoperative antibiotics were used after LC for acute calculous cholecystitis.
Wang et al. [33]	2025	Meta-analysis	36 RCTs N = 9,386 3 RCTs for infective complications in AC 3 RCTs investigated SSI in AC	Both IC (pooled log RR: 0.37; 95% CI: −0.60 - −0.14; p = 0.0017) and SSI rates (pooled log RR: 0.30; 95% CI: −0.51 - −0.09; p = 0.01) were lower when antimicrobial prophylaxis was given for all patients undergoing LC regardless of risk. Subgroup analysis for those with AC revealed no significant difference in IC between prophylactic antibiotics and no antibiotics (pooled log RR: −0.34; 95% CI: −0.74 - 0.05; p = 0.09). Regarding SSI, no difference in the AC subgroup was noted (pooled log RR: -0.43; CI –0.91 - 0.05; p = 0.08).
Negi et al. [35]	2021	Randomised controlled trial	N = 216	No difference in SSI rates was noted in high-risk patients undergoing LC (p = 0.568). Multivariate analysis was unable to be performed for high-risk subgroups due to the total number of SSI being 7.
Kim et al. [36]	2017	Randomised controlled trial	N = 200	No SSIs were reported in either group for analysis in this single-surgeon study on patients undergoing LC regardless of risk.

Intraoperative Contamination Events

A number of intraoperative factors that are difficult to predict in patients undergoing cholecystectomy increase the chance of infection. Intraoperative bile spillage is consistently identified as a risk factor for postoperative infection. A prospective study of over 1,000 patients demonstrated a four-fold increase in SSIs when bile contamination occurred, with this risk amplified when the gallbladder contains pus, or there is empyema or gangrenous cholecystitis [19]. There are no studies that investigate the role of antimicrobials in LC where bile spillage has occurred, despite the association between contamination and infectious complications. Bile duct instrumentation, which is also recognised as associated with increased infection risk, also remains to be studied.

Antibiotic Selection and Timing in Laparoscopic Cholecystectomy

Appropriate selection, timing and duration of antimicrobial prophylaxis are critical determinants of effectiveness and represent core principles of antimicrobial stewardship in LC. The frequent microbiology associated with postoperative complications is typically polymicrobial, such as biliary, skin and in cases of contamination, enteric organisms [37]. The commonly isolated pathogens include Escherichia coli, Klebsiella, Enterococcus and anaerobes in cases of AC or bile spillage [37].

In elective low-risk LC, the incidence of bactibilia is low and the majority of postoperative infections are superficial SSIs. The microbiological profile has historically underpinned guideline recommendations favouring narrow-spectrum agents targeting skin flora, most commonly a single, pre-incision dose of a first-generation cephalosporins such as cefazolin [38]. However, as synthesised in this review, multiple studies have demonstrated that routine prophylaxis in this setting does not result in meaningful reduction in SSI rates compared with no antibiotic use.

In higher-risk clinical contexts, including AC, bile contamination, empyema or gangrenous disease, broader microbial exposure and higher rates of bactibilia may be encountered. In such settings, international guidelines emphasise tailoring antimicrobial choice to the anticipated microbial spectrum, severity of disease and local resistance patterns while avoiding unnecessary escalation or prolonged therapy in the absence of established infection [29,39]. The evidence demonstrates that the antibiotics should be administered within 60 minutes prior to incision [40,41]. Delayed or postoperative antibiotics beyond a single dose do not offer any additional benefit and can contribute to AMR, increased toxicity and superinfections [40,41].

International Guidelines

The international guidelines consistently recommend that prophylactic antibiotics are not required in low-risk LC. The current iteration of the American Society of Health-System Pharmacists (ASHP) guidelines, the Surgical Infection Society Guidelines and the Australian Therapeutic Guidelines all recommend the use of antimicrobial prophylaxis only in high-risk patients [8,39,42]. Interestingly, the ASHP also state that it is reasonable to consider giving antimicrobial prophylaxis to all patients undergoing LC, regardless of risk, as several high-risk features are not possible to determine before surgical intervention. For high-risk LC, guidelines generally favour the use of prophylaxis [37,38]. In managing AC, the Surgical Infection Society Guidelines recommend against continuing antibiotics postoperatively for mild-moderate AC and limiting the duration for severe cholecystitis to not extend beyond four days [38]. Both the endorsement of microbial prophylaxis and stopping antibiotics after surgery is echoed by the Australian Therapeutic Guidelines for management of AC [39]. For other high-risk groups undergoing elective LC, the Australian Therapeutic Guidelines only recommend antimicrobial prophylaxis for those patients deemed high-risk pre-operatively. Notably, there is no mention of intraoperative events perceived as high-risk, i.e. bile spillage, instrumentation, or prolonged operative time. Table 3 summarises comparisons of international guideline recommendations for antimicrobial use in LC, stratified by operative context and clinical risk.

**Table 3:** Comparison of international guideline recommendations for antimicrobial use in LC AC: acute cholecystitis; LC: laparoscopic cholecystectomy

Guideline	Low-risk elective LC	Acute cholecystitis	Intraoperative bile spillage/contamination	Key stewardship notes
ASHP Surgical Prophylaxis Guidelines	Does not recommend routine prophylaxis for low-risk elective LC.	Emphasises that higher risk features (e.g., emergency surgery, diabetes, age >70, prolonged duration, gallbladder rupture/conversion) increase SSI risk and should prompt consideration of antibiotics consistent with risk stratification.	Discusses gallbladder rupture/bile spillage as a risk factor context; evidence cited includes studies where accidental rupture/spillage did not clearly benefit from additional antibiotic dosing beyond standard approaches.	Reinforces single dose, pre-incision timing principles and tailoring to local susceptibility patterns for biliary pathogens.
Surgical Infection Society Guidelines	Recommends against postoperative antibiotics after elective LC for symptomatic cholelithiasis/uncomplicated disease.	Recommends against postoperative antibiotics after LC for mild–moderate AC; for severe AC, recommends a maximum of 4 days of antibiotics (and possibly shorter) after cholecystectomy/source control.	Does not recommend routine prolongation purely for intraoperative events; emphasises limiting duration and avoiding continuation after adequate source control unless ongoing infection is suspected.	Strong focus on avoiding postoperative continuation when not indicated and limiting duration in severe disease.
Tokyo Guidelines	Not primarily a prophylaxis guideline for elective LC; focuses on infectious biliary syndromes and severity-based antimicrobial therapy.	For AC, recommends antimicrobials only before and at the time of surgery and for severe AC, recommends 4–7 days once source control is achieved.	Notes extended therapy may be considered when intraoperative findings suggest severe pathology (e.g. perforation/necrosis) and links duration decisions to adequacy of source control and clinical course.	Emphasises severity stratification and shortening duration after source control, with longer courses reserved for specific complications (e.g., bacteraemia/complications).
Australian Therapeutic Guidelines	For laparoscopic biliary surgery, prophylaxis is recommended only if risk factors for postoperative infection are present.	Prophylaxis is recommended for AC.	Does not provide specifics for bile spillage/contamination; management is left to clinical judgement within the broader risk stratification and harm–benefit assessment.	Highlights single-dose prophylaxis for most procedures and limits postoperative prophylaxis.

Antimicrobial Stewardship Implications

The findings of this review highlight LC as a high-impact target for antimicrobial stewardship interventions within general surgery. In low-risk elective LC, the clinical benefit of antimicrobial prophylaxis is not consistently observed. However, the continued high use, coupled with high-volume procedures, generates substantial antimicrobial exposure. Given the high procedural volume of LC, even modest reductions in prophylaxis use could translate into substantial reductions in antimicrobial exposure at a population level. Avoiding antibiotics in this group aligns with stewardship principles by minimising selection pressure for AMR, reducing the risk of Clostridium difficile infection and preventing avoidable adverse drug effects without compromising patient outcomes [15,43]. Stewardship considerations in high-risk LC are more nuanced and are characterised by evidentiary gaps rather than overt overuse.

Limitations

This narrative review has several limitations that should be acknowledged. First, as a narrative rather than systematic review, study selection was not governed by a predefined protocol or formal risk-of-bias assessment, introducing potential selection bias. The absence of formal risk-of-bias assessment or certainty grading limits the ability to weigh individual studies by methodological quality, and conclusions are therefore based on consistency of findings across the literature rather than graded strength of evidence. This approach allowed synthesis across randomised trials, observational studies and international guideline documents, reflecting the heterogeneity of the existing literature. Second, the available evidence is heavily skewed towards low-risk elective LC and AC, with limited high-quality data for other commonly cited high-risk subgroups such as patients with diabetes, obesity, immunosuppression or prolonged operative duration. Much of the evidence informing these populations is derived from retrospective observational studies, limiting causal inference. Finally, there is substantial heterogeneity across studies in definitions of “high-risk”, antibiotic regimens used, timing and duration of prophylaxis and outcome reporting. SSI definitions, surveillance periods, and follow-up methods vary widely, complicating direct comparison and synthesis of results.

Practice considerations and future research

Across multiple RCTs, systematic reviews and meta-analyses, routine antimicrobial prophylaxis in low-risk elective LC has not consistently demonstrated a clinically meaningful reduction in SSI rates. Therefore, this may represent a potential target for antimicrobial stewardship initiatives. In high-risk LC, particularly AC, an individualised approach may be considered in higher-risk clinical contexts. While baseline infection risk is higher, current evidence does not consistently demonstrate benefit from routine perioperative or postoperative antibiotic prophylaxis in mild-to-moderate AC and prolonged postoperative antibiotic courses are not supported by available trial evidence and are generally discouraged in major guidelines. For patients with severe cholecystitis, empyema, gangrenous disease or significant bile contamination, antimicrobial use should align with existing guideline recommendations, focusing on appropriate agent selection and limiting duration to the minimum effective period. Institutional protocols and stewardship programs often emphasise preoperative risk stratification, avoidance of postoperative prophylaxis in uncomplicated cases and early cessation of antibiotics where ongoing infection is not evident. Education, audit and feedback may help reduce persistent variation in practice.

There remains a clear need for high-quality prospective research to address persistent uncertainties in high-risk LC. Future RCTs should focus on clearly defined high-risk subgroups beyond AC, including patients with diabetes, obesity, immunosuppression, advanced age and prolonged operative time, rather than pooling heterogeneous populations. Trials should also evaluate intraoperative risk factors such as bile spillage, gallbladder perforation and ductal instrumentation, which are not predictable preoperatively but are consistently associated with increased infection risk. Standardisation of outcome measures, including uniform definitions of SSIs and consistent follow-up periods, is essential to improve comparability across studies. Additionally, future research should incorporate antimicrobial stewardship and health economic outcomes, such as antibiotic exposure, length of stay, readmission rates and AMR patterns, alongside traditional surgical endpoints. Finally, integration of prospective registry data with pragmatic clinical trials may offer a feasible approach to studying rare infectious outcomes in this high-volume procedure. Strengthening the evidence base in these areas is critical to inform future guideline development and reduce ongoing variation in antimicrobial prescribing practices.

## Conclusions

LC is one of the most common surgical procedures performed and is considered the gold standard for managing symptomatic gallstone disease. Debate regarding the role of perioperative antimicrobial use persists, reflecting heterogeneity in study design, patient populations and outcome definitions across the literature. While the findings across individual studies and meta-analyses are not entirely uniform, the preponderance of contemporary evidence indicates that routine prophylaxis has not consistently been shown to confer a clinically meaningful reduction in SSIs in low-risk elective LC. In contrast, evidence in high-risk LC remains limited and heterogeneous, precluding definitive conclusions. Given the profound implications for AMR, health economics and patient outcomes from antibiotic overuse, there remains a need for large, robust RCTs in high-risk LC. Subsequent investigations should also prioritise considering how individual high-risk groups may benefit from antimicrobial use individually. While currently inconclusive, this review nevertheless encourages a thoughtful approach to antimicrobial use in LC.
